# Osteogenic Potential of Sheep Mesenchymal Stem Cells Preconditioned with BMP-2 and FGF-2 and Seeded on an nHAP-Coated PCL/HAP/β-TCP Scaffold

**DOI:** 10.3390/cells11213446

**Published:** 2022-10-31

**Authors:** Sandra Stamnitz, Agnieszka Krawczenko, Urszula Szałaj, Żaneta Górecka, Agnieszka Antończyk, Zdzisław Kiełbowicz, Wojciech Święszkowski, Witold Łojkowski, Aleksandra Klimczak

**Affiliations:** 1 Laboratory of Biology of Stem and Neoplastic Cells, Hirszfeld Institute of Immunology and Experimental Therapy, Polish Academy of Sciences, R. Weigla 12, 53-114 Wroclaw, Poland; 2 Laboratory of Nanostructures and Nanomedicine, Institute of High Pressure Physics, Polish Academy of Sciences, Sokolowska 29/37, 01-142 Warsaw, Poland; 3 Division of Materials Design, Faculty of Materials Science and Engineering, Warsaw University of Technology, 141 Woloska Str., 02-507 Warsaw, Poland; 4 Department of Surgery, Faculty of Veterinary Medicine, Wroclaw University of Environmental and Life Sciences, pl. Grunwaldzki 51, 50-366 Wroclaw, Poland

**Keywords:** mesenchymal stem cells, scaffold, tissue engineering construct, bone regeneration

## Abstract

Mesenchymal stem cells (MSCs) attract interest in regenerative medicine for their potential application in bone regeneration. However, direct transplantation of cells into damaged tissue is not efficient enough to regenerate large bone defects. This problem could be solved with a biocompatible scaffold. Consequently, bone tissue engineering constructs based on biomaterial scaffolds, MSCs, and osteogenic cytokines are promising tools for bone regeneration. The aim of this study was to evaluate the effect of FGF-2 and BMP-2 on the osteogenic potential of ovine bone marrow-derived MSCs seeded onto an nHAP-coated PCL/HAP/β-TCP scaffold in vitro and its in vivo biocompatibility in a sheep model. In vitro analysis revealed that cells preconditioned with FGF-2 and BMP-2 showed a better capacity to adhere and proliferate on the scaffold than untreated cells. BM-MSCs cultured in an osteogenic medium supplemented with FGF-2 and BMP-2 had the highest osteogenic differentiation potential, as assessed based on Alizarin Red S staining and ALP activity. qRT-PCR analysis showed increased expression of osteogenic marker genes in FGF-2- and BMP-2-treated BM-MSCs. Our pilot in vivo research showed that the implantation of an nHAP-coated PCL/HAP/β-TCP scaffold with BM-MSCs preconditioned with FGF-2 and BMP-2 did not have an adverse effect in the sheep mandibular region and induced bone regeneration. The biocompatibility of the implanted scaffold-BM-MSC construct with sheep tissues was confirmed by the expression of early (collagen type I) and late (osteocalcin) osteogenic proteins and a lack of an elevated level of proinflammatory cytokines. These findings suggest that FGF-2 and BMP-2 enhance the osteogenic differentiation potential of MSCs grown on a scaffold, and that such a tissue engineering construct may be used to regenerate large bone defects.

## 1. Introduction

Bone, as a particularly dynamic connective tissue, undergoes continuous remodeling, during which osteoclasts remove damaged bone cells, then osteoblasts take part in the formation of new bone tissue. This process enables optimal adaptation of the bone structure to functional demands [1]. Although bone tissue has a tremendous regenerative capacity and is able to heal itself without fibrotic scar formation (contrary to most other tissues in the human body), approximately 5–10% of fractures are prone to healing abnormally [2]. Indeed, bone disorders are a daily occurrence in today’s clinical practice, contributing to a health, economic, and social burden on our aging population [3]. The most challenging pathological conditions are large traumatic bone damage and extensive bone loss due to tumor resection or failed surgery [4]. The clinical gold standard in orthopedic surgery is the autologous bone graft. However, there are limitations, such as deficient bone supply and donor site morbidity, that make it necessary to look for alternative methods. Although allografts or xenografts admittedly solve these problems, the drawbacks of these methods are immune reactions, risk of infectious agent transmission, high costs, and donor scarcity [5]. Therefore, more efficient alternatives to bone grafting are needed.

Technological advances have provided orthopedic implants made of biomaterials that reconstruct the damaged bone. These bioactive materials are able to elicit controlled action and reaction within the biological environment. However, biomaterials alone only serve as guidance for bone tissue, rather than as an osteoinductive agent, which means that immature cells are not recruited or stimulated to differentiate into osteogenic cells. In order to ensure not only structural but also functional integrity in complex skeletal defects, tissue engineering constructs composed of cells, scaffolds, and bioactive factors are required [6]. Mesenchymal stem cells (MSCs) are useful in the field of regenerative medicine because of their (1) multiple differentiation capability, (2) availability in large amounts, (3) usability in both autologous and allogenic transplants, (4) painless isolation methods, and (5) conformity with Good Manufacturing Practice guidelines (GMP) [7]. In addition to the capacity of MSCs to differentiate into bone tissue cells, they are also able to secrete bioactive factors, which impact other cells by regulating their significant biological functions, such as migration, proliferation, communication, and differentiation. The MSC secretome may be affected by specific culture conditions, such as medium composition or biomaterial properties [8]. Therefore, growth factors, such as fibroblast growth factor 2 (FGF-2) and osteogenic differentiating factors, such as bone morphogenetic proteins (BMPs), can be used to enhance the MSC osteogenic potential. BMPs and FGFs are among the main regulators in the bone healing cascade [9]. Although FGFs do not directly stimulate osteogenic differentiation, they modulate it by increasing osteoblast proliferation as well as inducing angiogenesis in the bone defect site [10]. BMPs are currently considered to be the proteins with the most beneficial effect on the repair of large bone defects [11]. They are involved in skeletal development and the osteogenic differentiation of MSCs and bone formation [12]. Furthermore, the Food and Drug Administration (FDA) has approved BMP-2 for clinical use in bone fracture treatment [13]. FGF-2 and BMP-2 were found to display a synergistic effect on MSC osteogenic differentiation [14,15,16].

In bone tissue engineering, scaffolds with osteoinductive and osteoconductive properties have been designed to provide 3D culture conditions for MSCs and enhance their regenerative properties, resulting in bone tissue formation [17]. Scaffolds play a crucial role in bone regeneration, acting as the extracellular matrix, which enables cells to grow in a three-dimensional environment and interact with other cells [18]. A safe and successful clinical application of biomaterials requires a scaffold with the appropriate properties, including biocompatibility, osteogenic stimulation, mechanical properties, and no immune response while being implanted into the host bone [19,20]. Biodegradable scaffolds consisting of composites, such as polymers combined with ceramics, display better physicochemical properties than non-composite scaffolds. For example, FDA-approved polycaprolactone (PCL) combined with ceramics, such as tricalcium phosphate (TCP) and hydroxyapatite (HA), shows both the controlled degradation kinetic potential of polymers and the considerable bioactive potential of ceramics [21].

Although many studies have been conducted in the field of bone regeneration, further investigation is needed for successful human clinical trials. In particular, the optimization of MSC culture methods on an appropriate scaffold and stimulation with bioactive factors in order to obtain a tissue engineering construct with optimal bone healing properties is required for the reconstruction of large bone tissue defects. It is worth mentioning that selecting an appropriate animal model for in vivo research is crucial in order to provide proof of concept for new bone tissue engineering techniques. Rodents are the most commonly used animal model. However, large animal models are more advantageous due to their body weight, bone size, and structural similarity to humans [22]. An adequate simulation of the biomechanics of human large bone defects based on animal models, such as mice or rats, is impossible. In addition, immunological responses in large animal models are closer to human responses [23] than in small animal models. Therefore, to mimic human clinical settings, the use of large animal models is absolutely essential [24].

Our previous study demonstrated that FGF-2 and BMP-2 enhance the osteogenic potential of sheep bone marrow-derived MSCs (BM-MSCs) in vitro [25]. In this study, we developed a bone tissue engineering construct consisting of an ovine BM-MSC and a nanohydroxyapatite-coated polycaprolactone/hydroxyapatite/β-tricalcium phosphate (nHAP-coated PCL/HAP/β-TCP) scaffold. We investigated the induced osteogenic effect of the bioactive factors FGF-2 and BMP-2 on this construct in vitro, after which we applied the construct in vivo to test its biocompatibility and regenerative potential in a large animal sheep model.

## 2. Materials and Methods

### 2.1. Biomaterial Characterization

#### Fabrication of a 3D Printed Scaffold

Composite scaffolds were tested. The composite material for scaffold fabrication was prepared from poly(ε-caprolactone) (PCL, PURASORB PC12, Corbion, Amsterdam, the Netherlands), hydroxyapatite (HAP, Ca5(OH)(PO4)3, Merck, Kenilworth, NJ, USA) and tricalcium phosphate (TCP, powder < 4 µm, 100% Beta-TCP, Ca3(PO4)2, Progentix) using the solvent casting method as with the previous studies [26,27,28]. The mass ratio of PCL:HAP:TCP was 9:4.5:4.5, respectively. The scaffolds were fabricated with a BioScaffolder printer (SYSENG, Germany) at a temperature of 110 °C using the G23 nozzle (ID 0.33 mm). The layers were printed with the 0°–90° pattern and a 0.27 mm layer thickness. The distance between the parallel fibers was set at 1.1 mm. Lastly, the obtained fiber diameter was 342.9 ± 19.5 µm, and the pore size was 718 ± 19.8 µm. The fabricated scaffolds had a cylindrical shape with a diameter of 6 mm and a height of 3 mm. The scaffolds were additionally coated with nano-hydroxyapatite (GoHAP), as previously described [29]. Nano-hydroxyapatite GoHAP Type 3 was produced by the Institute of High Pressure Physics, PAS, was used to form layers on the surface of the scaffolds (GoHAP nano-hydroxyapatite layers) [29,30,31,32]. Nano-hydroxyapatite GoHAP is a synthetic bone mineral that is highly similar to the hydroxyapatite naturally present in the bones. The mean particle size of GoHAP was 16 +/− 3 nm, the specific surface was 140 +/− 15 nm, and the Ca/P ratio was 1.61. The GoHAP nano-hydroxyapatite layers were deposited on the 3D-printed scaffolds using the sonocoating method described in [33,34]. The morphology of the nano-hydroxyapatite GoHAP layers was tested using a SEM (Ultra Plus, Carl Zeiss Meditec AG, Jena, Germany) with a secondary electron (SE) detector. The thickness of the layer shown in the SEM images was measured using the ImageJ software, version 1.53k. The number of individual layer thickness measurements was 68. As a control, pristine PCL scaffolds were fabricated at a temperature of 100 °C. All scaffolds were sterilized with 25 kGy of a Cobalt 60 gamma radiation source before the in vitro and in vivo experiments.

### 2.2. Sheep Bone Marrow-Derived Stem Cell (BM-MSC) Cultures on the Scaffold

The BM-MSCs collected from the same sheep were used in in vitro research and as autologous cells in in vivo experiments. BM-MSCs were isolated from the ovine iliac crest as previously described. A phenotype analysis of the sheep BM-MSCs confirmed the presence of specific MSC surface markers: CD73^+^, CD90^+^, and CD105^+^, as introduced in our previous paper [25]. In this study, BM-MSCs were thawed at passage 1 or 2, seeded for expansion, and then used for both in vitro and in vivo experiments at passage 2 or 3. Cells were cultured in the Minimum Essential Medium α-transformation, αMEM (IIET PAS, Wroclaw, Poland), supplemented with 10% fetal bovine serum, FBS (Biowest, Riverside, Montana, MT, USA, cat. no. S1810-500), 1% penicillin/streptomycin (Merck, Saint Louis, MO, USA, cat. no. P0781), 2 mM L-glutamine (Biowest, Riverside, Montana, MT, USA, cat. no. X0550-100) and incubated at 37 °C in 5% CO_2_ and 95% air. The medium was replaced two times a week. Prior to the in vitro cell seeding, the scaffold was placed in an appropriate culture plate well and washed with the culture medium for 30 min. Next, the medium was aspirated and the trypsinized BM-MSCs were seeded at a density of 5 *×* 10^4^ in 20 μL of the medium (for experiments lasting 21 days) or a 5 × 10^5^ cells/scaffold for the MTT and Picogreen assay. The cells were incubated for one hour at 37 °C to allow diffusion into and adhesion to the scaffold before the addition of the culture medium to each well. The experiments were conducted in three different medium conditions: (1) αMEM (control), (2) αMEM supplemented with 20 ng/mL FGF-2 (Merck, Saint Louis, MO, USA, cat. no. F0291), and (3) αMEM supplemented with 20 ng/mL FGF-2 and 100 ng/mL BMP-2 (Stem Cell Technologies, Grenoble, France, cat. no. 78004.1).

### 2.3. DAPI Staining for the Observation of BM-MSC Adhesion on a Scaffold

For the recognition of cell attachment to the scaffold, the DAPI staining method was used. BM-MSCs at a density of 5 × 10^4^ cells/scaffold were seeded onto scaffolds in a 48-well plate and cultured for 21 days in 500 μL of the αMEM medium supplemented with 20 ng/mL FGF-2 only or 20 ng/mL FGF-2 and 100 ng/mL BMP-2, and in an osteogenic differentiation medium (PromoCell, Heidelberg, Germany, cat. no. C-28013) with FGF-2 only or FGF-2 and BMP-2. The media were changed every three days. After three weeks, the culture media were aspirated, and the cell-scaffold constructs were washed with PBS (IIET PAS, Wroclaw, Poland) and fixed with 3.7% formaldehyde (Merck, Saint Louis, MO, USA, cat. no. 104003) for 30 min. Excess formalin was removed, and the scaffolds were washed again in PBS. Next, the cell nuclei were stained with DAPI (Vector Labs, Burlingame, CA, USA, cat. no. H-1200) for 20 min in the dark at RT. The scaffolds were rinsed three times with PBS to remove excess DAPI, and the cells were visualized using an Axio Observer fluorescence microscope (Zeiss, Jena, Germany).

### 2.4. Quantification of BM-MSCs on the Scaffold

To determine the effect of medium supplementation with FGF-2 only or with a combination of FGF-2 and BMP-2 on the capacity of the cells to adhere to the scaffold, the cells were quantified on the scaffold using the Picogreen solution from the Quant-it Picogreen kit (Invitrogen Life Technologies, France, cat. no. P7589), which binds double-stranded DNA. First, scaffolds in duplicates were placed in the V-bottom wells of a 96-well plate and incubated for 30 min at 37 °C with 200 μL of the αMEM medium (control) or with FGF-2 only or with FGF-2 and BMP-2. Next, the medium was aspirated, and 5 × 10^5^ cells in 20 μL of the medium with or without cytokines were seeded onto the scaffolds. After three hours of incubation, the scaffolds with the adhered cells were removed. To the remaining BM-MSCs at the bottom of the V-shape wells, 200 μL of a lysis solution was added, containing the Tris-EDTA buffer (Sigma-Aldrich, St. Louis, MO, USA, cat. no. T9285-100ML), 0.1% Triton X-100 (Sigma-Aldrich, St. Louis, MO, USA, cat. no. T8787-50ML), and 0.2 mg/mL proteinase K (Ambion RNA by Life Technologies, Waltham, MA, USA, cat. no. AM2548). The suspensions were incubated at 52 °C overnight, after which they underwent three heat shocks at −80 °C and RT, followed by sonication for 10 min. Meanwhile, a standard cell range from 0 to 5 × 10^5^ of BM-MSCs was prepared. DNA samples from the cell standard range and non-adherent cells were labelled with Picogreen in a 96-well plate and incubated for 10 min in the dark. Fluorescence was read at 520 nm using an EnSpire 2300 Multilabel Reader (Perkin Elmer LAS, Waltham, MA, USA). To quantify the cells, a standard curve was used. The experiment was repeated three times.

### 2.5. BM-MSC Viability on the Scaffold

The viability of BM-MSCs cultured with FGF-2 only or with a mixture of FGF-2 and BMP-2 on the scaffolds was analyzed using the MTT assay. The cells were seeded in duplicate at a density of 5 × 10^5^ BM-MSCs/scaffold in 20 μL of the medium in 96-well plates. The cells were allowed to attach to the scaffold for the first hour, after which 200 μL of the medium was added. Cell proliferation was analyzed after 1, 3, 5, and 7 days. Next, 20 μL of a 4 mg/mL MTT solution (Merck, Saint Louis, MO, USA, cat. no. M2128) were added. After incubation, over the next 4 h at 37 °C, the medium was aspirated, and 200 μL of DMSO (POCh, Gliwice, Poland, cat. no. 363550117) was added to solubilize the purple formazan crystals. Absorbance was quantified at 570 nm with a Wallac Victor2 microplate reader (Perkin Elmer LAS, Waltham, MA, USA). The medium in each well was replaced every two days. The MTT assay was repeated three times.

### 2.6. Alizarin Red S Staining Quantification

Alizarin Red S staining was performed to evaluate the osteogenic differentiation of BM-MSCs untreated or treated with FGF-2 only or with a combination of FGF-2 and BMP-2 and seeded on a scaffold. BM-MSCs at a density of 5 × 10^4^/scaffold were seeded in 20 μL of the medium in 96-well plates. After one hour of incubation, 200 μL of an osteogenic differentiation medium (PromoCell, Heidelberg, Germany, cat. no. C-28013) alone or supplemented with FGF-2 only or with FGF-2 and BMP-2 was added. The media were refreshed three times a week. After 7, 14, and 21 days of incubation, the differentiation potential was quantified based on Alizarin Red S staining using the cetylpyridinium chloride, CPC (Sigma-Aldrich, St. Louis, MO, USA, cat. no. C0732-100G) extraction method. For staining, the differentiation medium was removed, and the BM-MSC-scaffold constructs were washed with PBS and fixed for 20 min at RT in 3.7% formaldehyde. Next, the formaldehyde was aspirated, and the constructs were washed with PBS again and stained with 200 μL Alizarin Red S for 10 min at RT. For CPC extraction, PBS was removed from the wells, and the cells on the scaffold were incubated for two hours at 37 °C with 200 μL of a 10% CPC solution. The dye was transferred to a new 96-well plate and read at 405 nm with a Wallac Victor2 reader.

### 2.7. Alkaline Phosphatase Activity

To measure ALP activity, p-nitrophenyl phosphate, pNPP (Sigma-Aldrich, St. Louis, MO, USA, cat. no. P7998-100ML), was used as an ALP substrate. BM-MSCs were cultured on a scaffold following the same procedure as in the Alizarin Red S staining Section 2.6. On days 7, 14, and 21 of incubation, the BM-MSC-scaffold constructs were washed with PBS, and 200 μL of pNPP were added. After one hour of incubation at 37 °C, the yellow product was transferred to a fresh 96-well plate in order to measure absorbance at 405 nm using a Wallac Victor2 reader.

### 2.8. qRT-PCR for Osteogenic Gene Expression

To analyze the impact of FGF-2 and BMP-2 on the osteogenic gene expression of the tissue engineering construct consisting of an nHAP-coated PCL/HAP/β-TCP scaffold and BM-MSCs, the constructs were cultured for 21 days in a 6-well plate in the αMEM control medium, or supplemented with FGF-2 only or with FGF-2 and BMP-2. The samples were collected after 7, 14, and 21 days into 2 mL Matrix M tubes with beads (MP Biomedicals, Solon, OH, USA, cat. nos. 6923050 and 6540034) with 1 mL of the TRIzol reagent (Ambion RNA by Life Technologies, Waltham, MA, USA, cat. no. 15596026) and homogenized using a FastPrep-24 tissue and cell homogenizer (MP Biomedicals, Solon, OH, USA). Total RNA was extracted and purified with the NucleoSpin RNA Kit (Macherey-Nagel, Düren, Germany, cat. no. 740955.50) according to the manufacturer’s instructions for RNA purification in combination with TRIzol lysis. A reverse transcription of total RNA in the amount of 1 μg from each sample was conducted using the RevertAid First Strand cDNA Synthesis Kit (Thermo Fisher, Vilnius, Lithuania, cat. no. K1622). Real-time PCR was performed with the Power SYBR Green PCR Master Mix (Life Technologies, Warrington, UK, cat. no. 4367659), and the rate of dye incorporation was analyzed using the ViiA 7 Real-Time PCR System (Applied Biosystems, Foster City, CA, USA). The reactions were carried out three times with two biological replicates with the following program settings: initial denaturation at 95 °C for 10 min, followed by 40 cycles of denaturation at 95 °C for 15 s, annealing at the Tm (°C) of the primers listed in Table 1 for one minute, and extension at 72 °C for 40 s. The levels of the housekeeping gene GAPDH transcript were used to normalize all PCR product quantifications (ΔCT), and the relative mRNA expression level was obtained using the 2^−∆∆CT^ calculation method.

### 2.9. Animal Surgery

The study was approved by the local Animal Ethics Committee at the Institute of Immunology and Experimental Therapy PAS (no. 63/2017). Preliminary research in vivo using four adult sheep weighing from 42 to 54 kg was performed primarily to assess the biocompatibility and osteogenic potential of the scaffold-BM-MSCs construct in the mandibular region of a large animal model. Moreover, this region was used to assess the feasibility of the treatment method for critical-sized mandibular bone defects. In the small ruminant model, the mandibular angle is the optimal site to create artificial, critical bone defects due to the specific anatomy and physiology of this species. Creating the defect more cranially would damage the tooth roots, disrupting the physiological chewing process and leading to the animal’s death. All surgical procedures were performed by experienced surgeons at the Department and Clinic of Surgery, Faculty of Veterinary Medicine, Wroclaw University of Environmental and Life Sciences.

#### 2.9.1. Anesthesia and Analgesia

All sheep received medetomidine (0.01 mg/kg, Cepetor, CP-Pharma Handelsges), butorphanol (0.1 mg/kg, Butomidor Richter Pharma AG, Oberösterreich, Austria), and a combination of tiletamine and zolazepam (2 mg/kg, Zoletil 100, Virbac, Carros, France) intramuscularly (im). General anesthesia was induced with propofol (Propofol-Lipuro^®^, 10 mg/mL, B. Braun Melsungen AG, Melsungen, Germany) at an initial dose of 2 mg/kg to effect, in order to permit tracheal intubation. The anesthesia was maintained with isoflurane (IsoVet, Piramal Healthcare, Morpeth, UK) in oxygen. After a bolus of fentanyl (Fentanyl WZF, Polfa Warszawa, Poland; 3 mcg/kg), analgesia was continued with a constant rate infusion of fentanyl (CRI—0.3 mcg/kg/min). Postoperative pain was managed with meloxicam (0.2 mg/kg sc, Metacam, Boehringer Ingelheim Vetmedica GmbH, Ingelheim am Rhein, Germany), metamizole (50 mg/kg iv, Injectio Pyralgini Biowet Puławy, Biowet Puławy Sp. z o.o., Puławy, Poland), and buprenorphine (0.02 mg/kg im, Bupaq Multidose, Richter Pharma AG, Austria). Intravenous fluids (crystalloids) were given in each case at a rate of 10 mL/kg/h. Throughout the surgical procedure, the vital parameters of the animals (heart rate, respiratory rate, end-tidal CO_2_, blood pressure, oxygen saturation, and temperature) were monitored continuously (Datex-Ohmeda S5 monitor, Helsinki, Finland).

#### 2.9.2. Surgical Procedure for Scaffold Implantation

**Figure 1:**
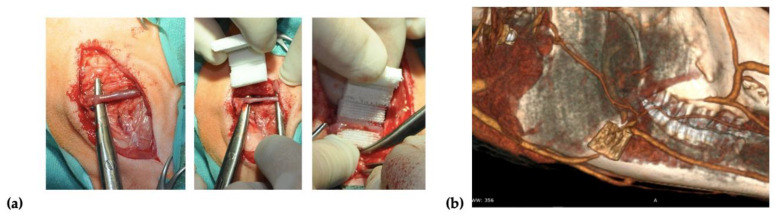
(**a**) Scaffold implantation in the masseter muscles, around the facial vein. (**b**) A tomography scan performed immediately after the implantation procedure.

Two incisions of up to 8 cm in length were made on the right and left side over the mandibular surface. Before the scaffold implantation, 20 *×* 10^6^ of autologous BM-MSCs treated with FGF-2 (*n* = 2) or FGF-2 and BMP-2 (*n* = 2) were resuspended in 2 mL of 0.9% NaCl and seeded on each scaffold using a 3-mL syringe with a 27-gauge needle. After dissecting through the masseter muscle, a facial vein was isolated, and the scaffold was implemented around the vessel in a manner that enabled blood to flow through the vessel (Figure 1a). To confirm the location of the scaffold and patency of a blood vessel, a computed tomography (CT) scan (Siemens Somatom Emotion 16) with iodine-based contrast (Iomeron 400, Bracco Imaging Deutschland GmbH, Konstanz, Germany) was performed after the implantation (Figure 1b). After the surgery, the sheep were observed for a period of 6 months. Wound healing, the general health of the animals, and signs of inflammation were monitored.

### 2.10. Collagen Type I and Osteocalcin Immunofluorescence Staining

Sections prepared from the nHAP-coated PCL/HAP/β-TCP scaffolds implanted with BM-MSCs treated with FGF-2 alone (*n* = 2) or FGF-2 and BMP-2 (*n* = 2) were retrieved from recipient sheep 6 months after surgery. The formalin-fixed paraffin-embedded slides were placed in a 60 °C oven for an hour. Next, the sections were deparaffinized with xylene (CHEMPUR, Piekary Sl., Poland, cat. no. 115208603) twice, and washed with 100% ethanol (CHEMPUR, Piekary Sl., Poland, cat. no. 113964800), 70% ethanol, and 40% ethanol, every wash lasting 10 min. After the washes, the sections were immersed in miliQ water. Antigens were retrieved using a 10 mM sodium citrate buffer, pH 6.0 (IIET PAS, Wroclaw, Poland) for 20 min at 98 °C. Afterwards, the slides were cooled to RT, washed twice with PBS, and proceeded with immunostaining. Each section was incubated overnight at 4 °C with 150 μL of 1:100 diluted primary antibody, rabbit anti-collagen type I (Abcam, Cambridge, UK, cat. no. AB34710), or mouse anti-osteocalcin (Abcam, Cambridge, UK, cat. no. AB13420). Next, each slide was washed with PBS three times and incubated for 30 min in the dark at RT with 150 μL of 1:500 diluted secondary goat anti-rabbit (Abcam, Cambridge, UK, cat. no. AB6717) or goat anti-mouse (Abcam, Cambridge, UK, cat. no. AB6785) FITC-conjugated solutions. After three PBS washes, DAPI (Vector Labs, Burlingame, CA, USA, cat. no. H-1200) was used for nuclei staining for 20 min of incubation in the dark at RT. Finally, the sections were washed with PBS and the immunofluorescence staining was visualized using an Axio Observer inverted fluorescence microscope (Zeiss, Jena, Germany) and analyzed using the Zeiss Zen Blue software, version 2.6.

### 2.11. Ovine Cytokine Array

To assess the biocompatibility of the implanted scaffold covered with BM-MSCs, the activity of trophic factors promoting osteogenesis and the expression of proinflammatory cytokines associated with the immune response were examined. Serum samples from the sheep undergoing the implantation of the nHAP-coated PCL/HAP/β-TCP scaffold covered with BM-MSCs and treated with FGF-2- (*n* = 2) or FGF-2 and BMP-2 (*n* = 2) were collected one week prior to and also one, two, and four weeks after surgery. The C-Series Ovine (Sheep) Cytokine Array C1 Kit (Ray-Bio^®^, Norcross, GA, USA, cat. no. AAO-CYT-1-8) was used to evaluate the relative level of cytokines in the sheep serum. Firstly, the samples were centrifuged at 3000 rpm for 10 min at RT and diluted five times with a blocking buffer, included in the Array Kit. The experiment was conducted according to the manufacturer’s protocol. The data were obtained with the Protein Array Analyzer plugin for the ImageJ software. The differences in the relative protein expression of the serum samples were presented on heat maps created using the GraphPad Prism version 7 (GraphPad Software, Inc., San Diego, CA, USA).

### 2.12. Statistical Analysis

Statistical analysis was performed with the GraphPad Prism 9 software, version 9.2.0. using one-way analysis of variance (one-way ANOVA) with Dunnett’s test for multiple comparison. A *p*-value < 0.05 was considered statistically significant.

## 3. Results

### 3.1. Biomaterial Characterization

**Figure 2:**
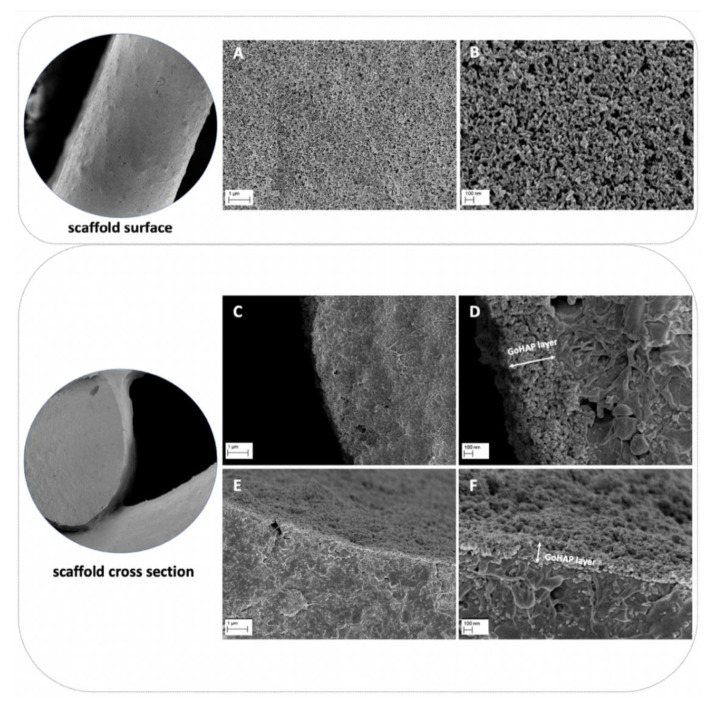
SEM images of the obtained nano-hydroxyapatite GoHAP layers on nHAP-coated PCL/HAP/β-TCP scaffolds: (**A**,**B**) outer fiber surface of the scaffold; (**C**–**F**) cross-section of the inner fibers of the scaffold.

The sonocoating method [33,34] allowed for the deposition of a bioactive layer consisting of nano-hydroxyapatite nanoparticles (GoHAP Type 3) on the surface of the 3D-printed scaffold. SEM imaging showed that the GoHAP nano-hydroxyapatite layers deposited on the scaffold surface were homogeneous. Moreover, the GoHAP nano-hydroxyapatite layers were present not only on the outer surface of the scaffold (Figure 2A,B), but also on the surface of the scaffold pores, as demonstrated by the cross-sectional imaging of the coated scaffold (Figure 2C–F). The average thickness of the GoHAP nano-hydroxyapatite layer was about 220 ± 70 nm.

### 3.2. BM-MSC Attachment and Spreading on the nHAP-Coated PCL/HAP/β-TCP Scaffold

**Figure 3:**
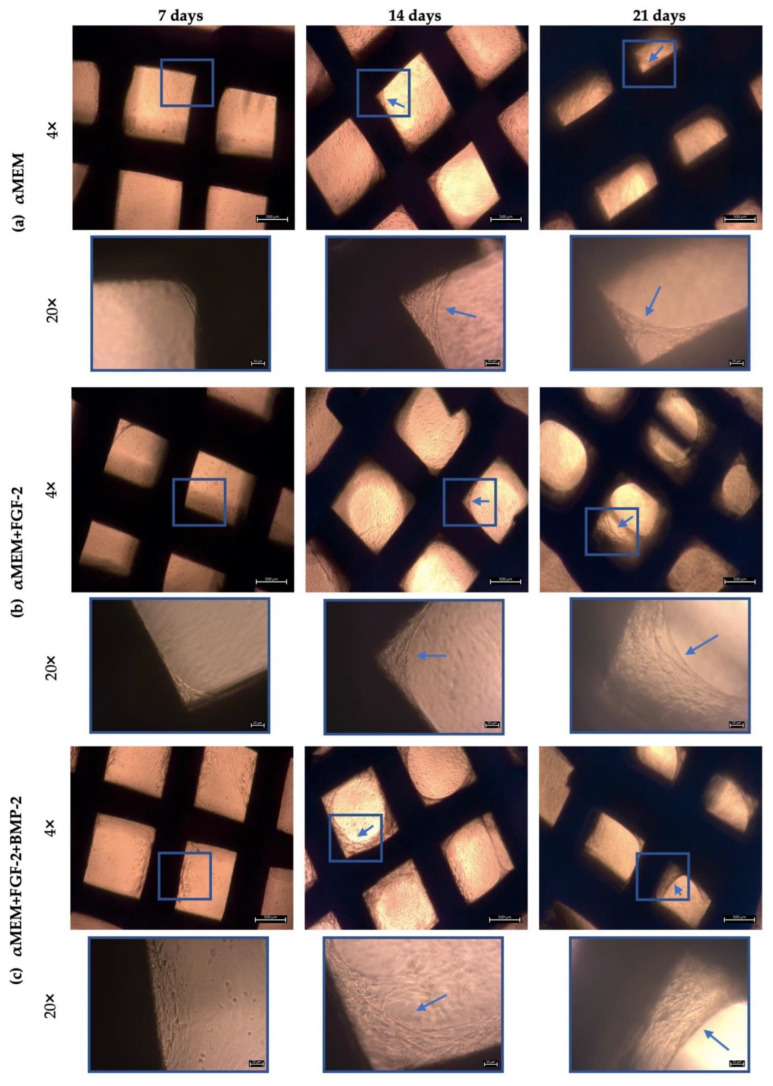
Morphology of sheep BM-MSCs on days 7, 14, and 21, cultured on the nHAP-coated PCL/HAP/β-TCP scaffold in different culture media. In response to FGF-2 and BMP-2 stimulation, the cells attached differently to the scaffold. (**a**) BM-MSCs cultured in αMEM started to grow over the space between the scaffold structures on day 14; marked with blue arrows in the picture. (**b**) FGF-2-treated BM-MSCs created a specific 3D structure on the scaffold on day 21. This 3D cell structure covered a larger area than with the untreated cells. (**c**) BM-MSCs stimulated with FGF-2 and BMP-2 had the best ability to adhere to the scaffold surface after 14 days of incubation and maintained this ability on day 21 of observation. Under each picture, a higher magnification (20×) was added to show the area marked with the blue frame.

The attachment and spread of BM-MSCs on the nHAP-coated PCL/HAP/β-TCP scaffold was observed microscopically at the following time points: 7, 14, and 21 days. The capacity for cell adhesion was compared between (a) the control medium αMEM, (b) αMEM supplemented with FGF-2, and (c) αMEM supplemented with FGF-2 and BMP-2. In all three media, the cells readily adhered to the scaffolds (Figure 3). Untreated BM-MSCs began to gather around the cross-bar segments of the scaffold after 14 days of incubation. However, by day 21, there were still large unfilled spaces between the scaffold bars (Figure 3a). In contrast, BM-MSCs cultured in αMEM and FGF-2 showed a more favorable adhesion to the scaffold. After 21 days, they covered a sizable space around the scaffold bars (Figure 3b). Nevertheless, the best capacity to attach and spread on the scaffold was observed for the cells treated with FGF-2 and BMP-2. Migrating BM-MSCs started to spread between the scaffold bars on day 7, and after 14 days, the cells covered a larger area between the bars compared to the cells cultured with FGF-2. However, on day 21, FGF-2- and BMP-2-treated cells filled the space between the scaffold bars to an extent comparable to that of FGF-2-treated BM-MSCs (Figure 3c). Together, these observations indicated that ovine BM-MSCs maintained good adhesion ability and material affinity on the nHAP-coated PCL/HAP/β-TCP scaffold and that pretreatment with FGF-2 and BMP-2 promoted this capacity.

### 3.3. BM-MSC Adhesion on the nHAP-Coated PCL/HAP/β-TCP Scaffold Analyzed with DAPI Staining

**Figure 4:**
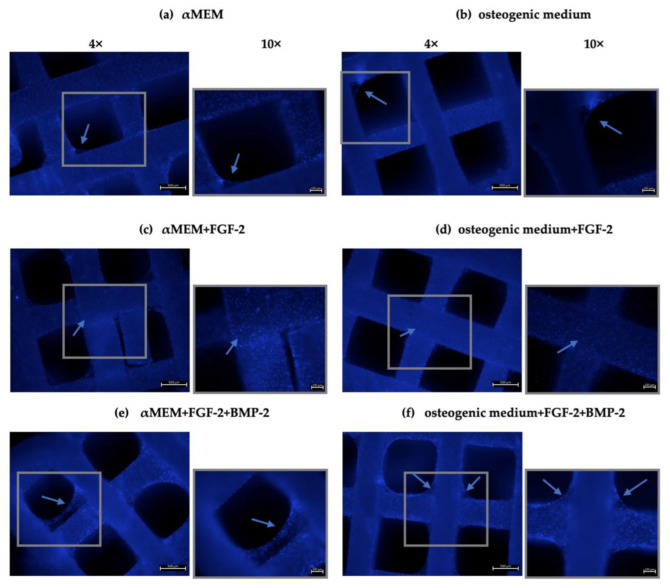
Ovine BM-MSC nuclei stained with DAPI (blue) on an nHAP-coated PCL/HAP/β-TCP scaffolds 21 days after seeding, cultured in (**a**) αMEM, (**b**) osteogenic medium, (**c**) αMEM + FGF-2, (**d**) osteogenic medium + FGF-2, (**e**) αMEM + FGF-2 + BMP-2 and (**f**) osteogenic medium + FGF-2 + BMP-2. The BM-MSCs maintained good material affinity on the scaffold, as indicated by the number of nuclei on the cross-bar segments of the scaffold. However, stimulation with FGF-2 and BMP-2 increased cell proliferation not only on the scaffold bars but also in the space between the bars. A higher magnification (10×) was added next to each picture in order to show the area marked with the gray frame.

nHAP-coated PCL/HAP/β-TCP scaffolds were seeded with BM-MSCs in a control medium αMEM and an osteogenic medium (Figure 4a,b) for 21 days. Furthermore, to investigate the impact of FGF-2 and BMP-2 on the ability of the cells to adhere to the scaffold, the culture media were supplemented with FGF-2 alone or FGF-2 and BMP-2. DAPI staining enabled the visualization of the cell nuclei on the scaffold bars, which are not visible with light microscopy. All analyzed culture media conditions promoted cell adhesion to the scaffold surface. The supplementation of FGF-2 seemed to not play a significant role in cell proliferation on the scaffold (Figure 4c,d). In contrast, supplementation of both the control medium and the osteogenic medium with both FGF-2 and BMP-2 stimulated the cells not only to proliferate on the scaffold surface but also to fill the space between the scaffold bars with a network of cells (Figure 4e,f).

### 3.4. Number of Cell on the Scaffold Analyzed with the Picogreen Assay

**Figure 5:**
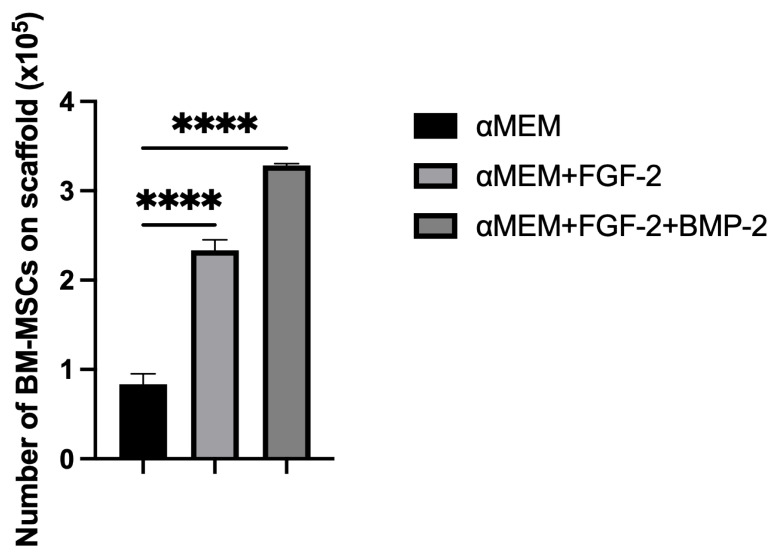
Number of BM-MSCs on an nHAP-coated PCL/HAP/β-TCP scaffold 3 h post-seeding in αMEM with or without FGF-2 alone or FGF-2 and BMP-2. Cells treated with FGF-2 adhered better to the scaffold than untreated cells. However, the highest number of cells that adhered to the scaffold was observed in the medium supplemented with both FGF-2 and BMP-2. The experiment was conducted in three independent assays, each in triplicate. **** *p* < 0.0001.

The adhesion capacity of BM-MSCs to the nHAP-coated PCL/HAP/β-TCP scaffold was evaluated through the quantification of total DNA, labelled with Picogreen. After 3 h of incubation of 5 × 10^5^ cells on the scaffold, the number of cells that adhered to the scaffold was assessed in the αMEM medium supplemented with only FGF-2 or FGF-2 and BMP-2 and compared to the control medium αMEM. The number of BM-MSCs cultured with FGF-2 alone or FGF-2 and BMP-2 on the scaffold was significantly higher compared to the control. In the control medium αMEM, out of the 5 × 10^5^ seeded cells, less than 10^5^ cells adhered to the scaffold, whereas in αMEM and FGF-2, over 2.5 × 10^5^ BM-MSCs attached to the scaffold. Nevertheless, supplementation with both FGF-2 and BMP-2 resulted in the highest adherence of the BM-MSCs to the scaffold, as indicated by the adhesion of 3.5 × 10^5^ cells to the scaffold (*p* < 0.0001, Figure 5).

### 3.5. Cell Proliferation and Viability

**Figure 6:**
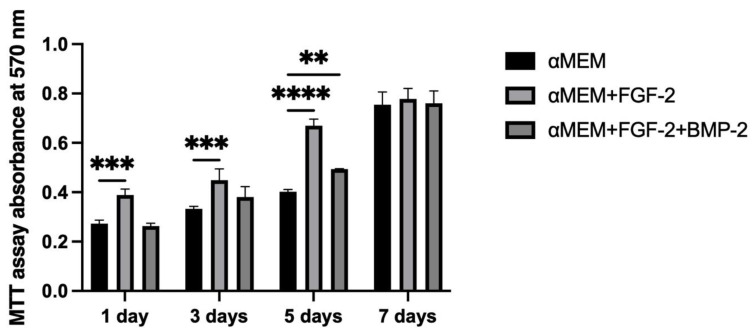
Ability of BM-MSCs to proliferate on the scaffold in αMEM supplemented with only FGF-2 or both FGF-2 and BMP-2, assessed with MTT over 7 days of incubation. The proliferation rate of the cells increased over time. The highest cell proliferation on the scaffold was observed for BM-MSCs treated with FGF-2 only, and the lowest proliferation was observed for untreated cells up to day 5. The MTT assay was performed in three independent experiments in triplicate each. ** *p* < 0.005, *** *p* < 0.001, **** *p* < 0.0001.

The impact of FGF-2 and BMP-2 on BM-MSCs proliferation and viability on the nHAP-coated PCL/HAP/β-TCP scaffold was evaluated with the use of an MTT assay. During 7 days of incubation, the proliferation rate of sheep BM-MSCs growing on scaffold increased over time, independently of the culture medium. These results indicates that nHAP-coated PCL/HAP/β-TCP scaffold is not toxic for the cells and stimulates cell proliferation. Nevertheless, FGF-2 was found to have the most beneficial effect on the BM-MSC proliferation capacity until day 5 of cell culture (*p* < 0.001, Figure 6). The addition of both FGF-2 and BMP-2 to the medium resulted in a greater proliferation rate compared to the untreated cells. However, the rate was not higher than in FGF-2-only treated cells. On day 7, the proliferation ability was almost the same in all the culture media.

### 3.6. Osteogenic Differentiation with Alizarin Red S Staining

**Figure 7:**
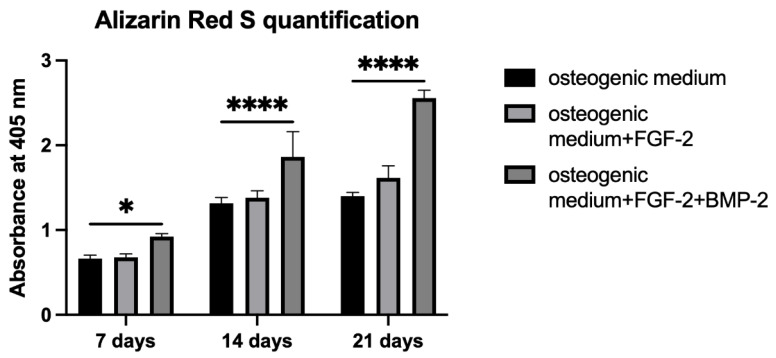
Quantification of Alizarin Red S staining of BM-MSCs growing on a scaffold using the CPC extraction method. Cells cultured in an osteogenic medium supplemented with both FGF-2 and BMP-2 had the greatest osteogenic differentiation potential compared to untreated or FGF-2-only treated cells. Alizarin Red S was quantified in three independent experiments in duplicate each. * *p* < 0.05, **** *p* < 0.0001.

A semi-quantitative analysis of the osteogenic differentiation capacity of BM-MSCs growing on the nHAP-coated PCL/HAP/β-TCP scaffold was conducted through CPC extraction of Alizarin Red S-stained mineral particles. The absorbance of the extracted solution was measured on days 7, 14, and 21 in an osteogenic medium with or without FGF-2 alone or with FGF-2 and BMP-2. The results showed that the osteogenic differentiation capacity increased over time in all culture conditions. Supplementation with FGF-2 only had a very low impact on osteogenesis after 21 days of cell treatment compared to control (Figure 7, absorbance at 405 nm 1.62 vs. 1.40). However, on day 21, supplementation with both FGF-2 and BMP-2 had the highest impact on BM-MSC osteogenesis compared to untreated and FGF-2-only treated cells (absorbance at 405 nm 2.56 vs. 1.40 and 1.62, *p* < 0.0001).

### 3.7. ALP Activity

**Figure 8:**
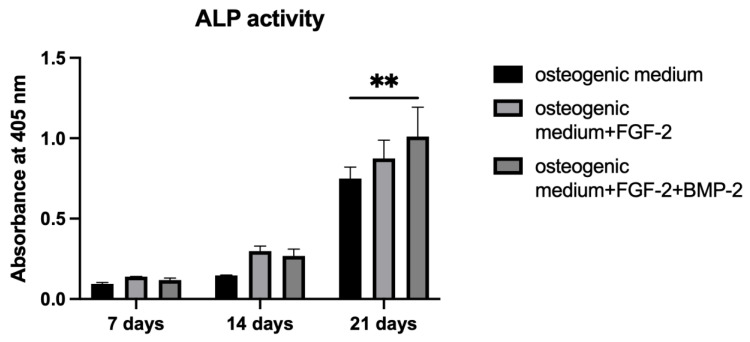
ALP activity of BM-MSCs grown on an nHAP-coated PCL/HAP/β-TCP scaffold treated or untreated with only FGF-2 or FGF-2 and BMP-2. ALP activity measured at 405 nm significantly increased for all culture conditions on day 21. The highest ALP activity was observed for FGF-2- and BMP-2-treated cells. The ALP assay was repeated in three independent experiments in duplicate each. ** *p* < 0.005.

An analysis of ALP activity showed that BM-MSCs growing on the scaffold required 21 days for a significant increase in the ALP rate, independently of culture media. Between 7 and 14 days of cell incubation, there were no significant differences in ALP activity across all groups. However, on day 21, ALP activity increased significantly, even for untreated cells, compared to 14 days of incubation (Figure 8, absorbance at 405 nm 0.75 vs. 0.15). Nonetheless, FGF-2 and BMP-2 stimulated osteogenic differentiation through an increase in ALP activity more than untreated cells (on day 21, absorbance 1.01 vs. 0.75, *p* < 0.005).

### 3.8. Expresssion of Osteogenic Genes in BM-MSC Grown on the nHAP-Coated PCL/HAP/β-TCP Scaffold Depending on FGF-2 and BMP-2 Stimulation

**Figure 9:**
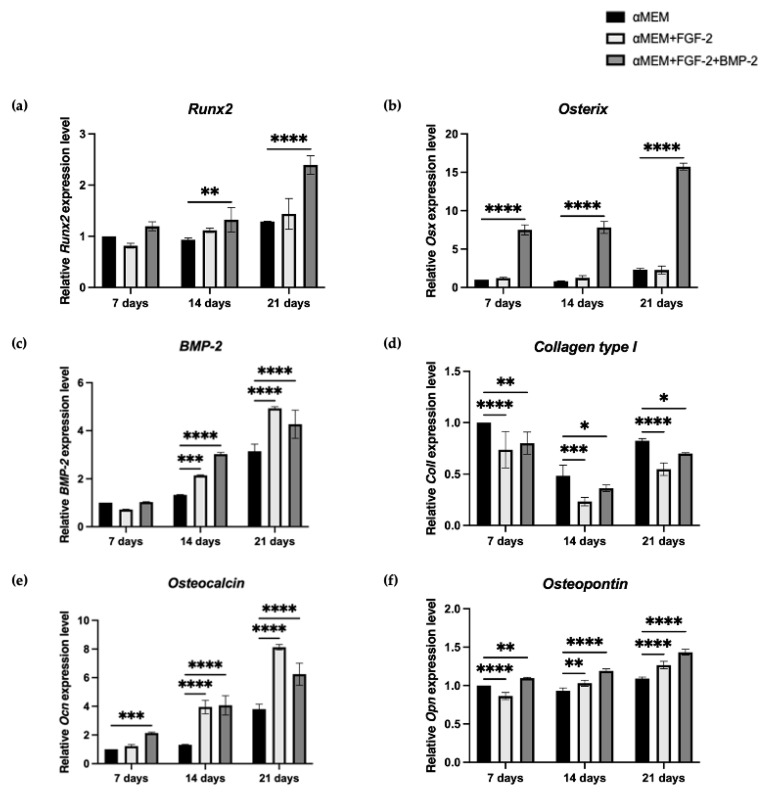
Relative expression of the early osteogenic differentiation gene markers (**a**) *Runx2*, (**b**) *osterix (Osx),* (**c**) *BMP-2,* and (**d**) *collagen type I (ColI)* and late osteogenic gene markers (**e**) *osteocalcin (Ocn)* and (**f**) *osteopontin (Opn),* analyzed with real-time PCR in ovine BM-MSCs grown on an nHAP-coated PCL/HAP/β-TCP scaffold with or without FGF-2 alone or with FGF-2 and BMP-2 for 21 days. Three independent experiments were each performed in duplicate. * *p* < 0.05, ** *p* < 0.005, *** *p* < 0.001, **** *p* < 0.0001.

On days 7, 14, and 21, following treatment with FGF-2 alone or FGF-2 and BMP-2, samples of sheep BM-MSCs grown on the nHAP-coated PCL/HAP/β-TCP scaffold were collected for real-time PCR. The influence of cytokines on BM-MSC osteogenic differentiation was evaluated based on the relative expression levels of early osteogenesis gene markers: *Runx2*, *osterix* (*Osx*)*, BMP-2*, and *collagen type I* (*ColI*) and late osteogenesis gene markers: *osteopontin* (*Opn*) and *osteocalcin* (*Ocn*). Relative expression of *Runx2* increased over time for BM-MSCs grown on the scaffold and treated with FGF-2 alone or FGF-2 and BMP-2. The highest peak was observed for the cells treated with FGF-2 and BMP-2 after 21 days (RQ 2.39 vs. 1.29 in control; *p* < 0.0001) (Figure 9a). For the untreated cells or cells treated with FGF-2 only, there was no significant difference in the relative expression of *Runx2,* and the expression was at a similar level regardless of the time-point of observation. Treatment with FGF-2 and BMP-2 significantly upregulated the mRNA level of *osterix* from day 14 to 21 (RQ 7.82 vs. 15.74) (Figure 9b), whereas between day 7 and 14, there was no significant difference (RQ 7.51 vs. 7.82). The relative expression of *osterix* for untreated or FGF-2-treated cells increased over time and was the highest after 21 days of incubation (RQ 2.31 and 2.28); however, it was much lower compared to FGF-2- and BMP-2-treated cells. Interestingly, the relative expression of *BMP-2* was upregulated over time in all cases; however, the highest peak was observed after 21 days for FGF-2-alone treated cells, and not for FGF-2- and BMP-2-treated BM-MSCs (RQ 4.92 vs. 4.28) (Figure 9c). Interestingly, on day 14, FGF-2- and BMP-2-treated cells showed the highest relative expression of *BMP-2* (RQ 3.03 vs. 1.33 in control; *p* < 0.0001). The relative mRNA expression of *collagen type I* decreased after 14 days of incubation in all culture conditions to slightly increase after 21 days; however, it was still lower than on day 7 for all groups (RQ on day 7, 14, and 21 for control 1.00, 0.48, and 0.83, respectively) (Figure 9d). Untreated BM-MSCs showed a higher expression of *ColI* than FGF-2 alone- or FGF-2- and BMP-2-treated cells across all analyzed time points. The relative expression level of the late osteogenic marker *osteopontin* increased over time in all groups. However, the increase in the control group was negligible, with the value remaining stable at an RQ of about 1.00 (Figure 9e). BM-MSCs grown on the nHAP-coated PCL/HAP/β-TCP scaffold with FGF-2 and BMP-2 for 21 days showed the highest expression of *Opn* (RQ 1.43 vs. 1.09 in control; *p* < 0.0001). Differences in the second late osteogenic gene expression marker, *osteocalcin*, were more prominent. On day 7, cells treated with FGF-2 and BMP-2 showed the highest relative expression of *Ocn* (RQ 2.13 vs. 1.00 in control; *p* < 0.001) (Figure 9f). On day 14, *Ocn* expression increased for all culture conditions and was similar for FGF-2 alone- or FGF-2- and BMP-2-treated cells (RQ 3.95 and 4.07). However, after 21 days, it was higher in BM-MSCs cultured with FGF-2 than in BM-MSCs treated with both FGF-2 and BMP-2 (RQ 8.13 vs. 6.24).

### 3.9. Clinical Assessment

After the implantation of the nHAP-coated PCL/HAP/β-TCP scaffolds, the animals were monitored for wound healing, general health, and signs of inflammation. The implanted scaffolds were well tolerated in all animals, with only minor side effects associated with wound healing, such as temporary swelling over two days after surgery. No fever or other undesired signs of inflammation were observed.

### 3.10. Immunofluorescent Staining of Osteocalcin and Collagen Type I

**Figure 10:**
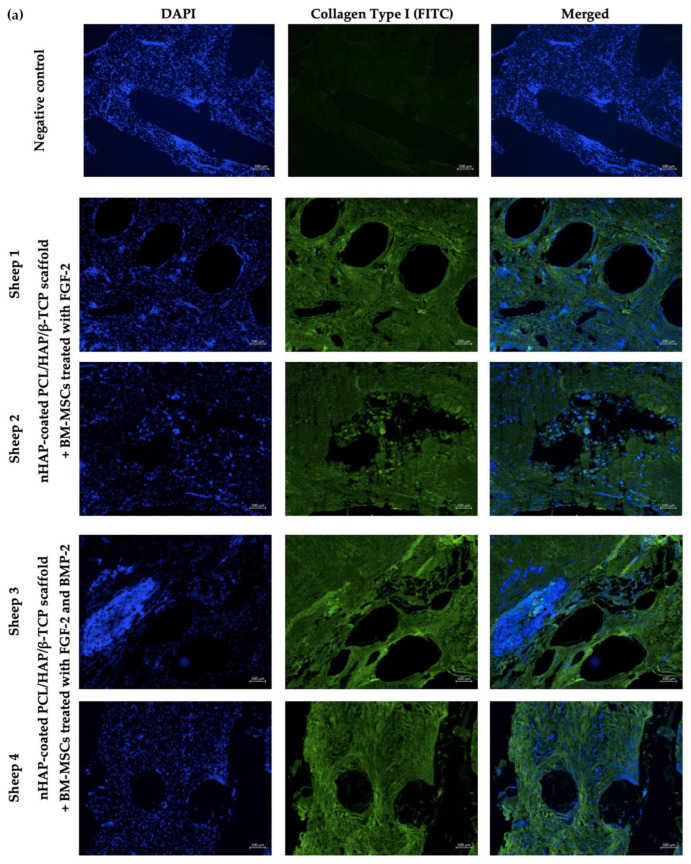

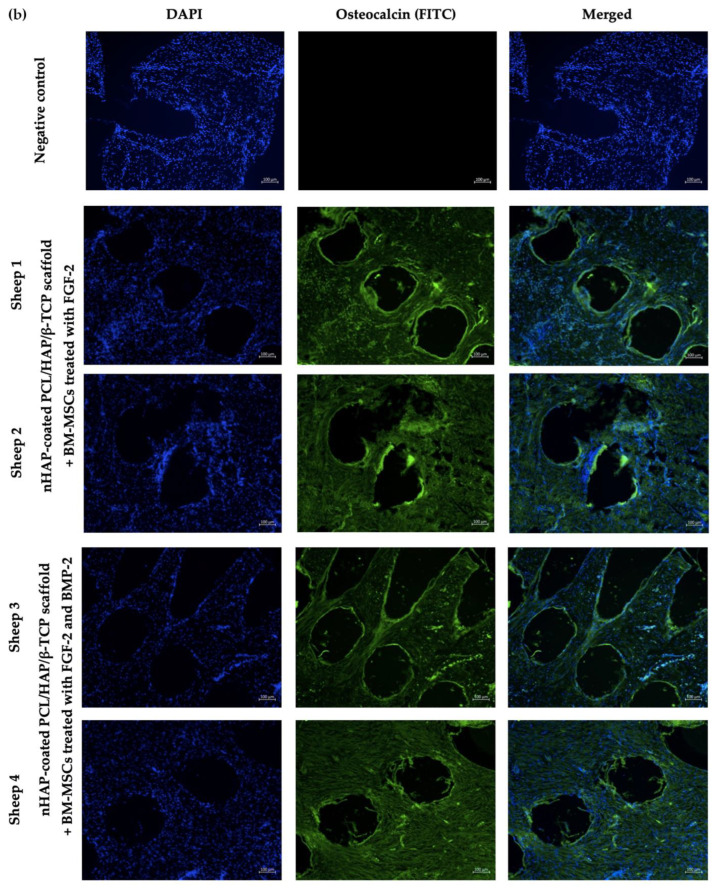

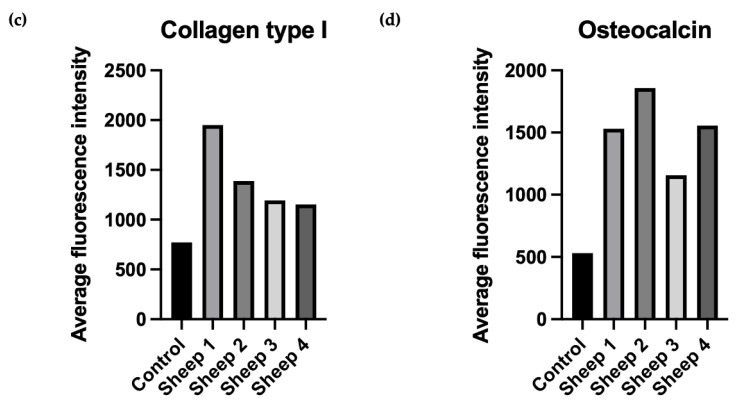
Immunofluorescence analysis of the early osteogenic marker collagen type I (**a**) and late osteogenic marker osteocalcin (**b**) for scaffold sections retrieved from sheep 6 months after the implantation of a scaffold and BM-MSCs treated with FGF-2 or FGF-2 and BMP-2. Merged: DAPI staining of the nucleus (blue) and antibody staining (green fluorescence). Intensity of fluorescence of collagen type I (**c**) and osteocalcin (**d**) compared to negative controls.

The in vivo osteogenic differentiation potential of the BM-MSCs treated with only FGF-2 or FGF-2 and BMP-2 together with the nHAP-coated PCL/HAP/β-TCP scaffold grafted into the ovine mandible was evaluated using immunofluorescent staining for the early osteogenic marker collagen type I and for the late osteogenic marker osteocalcin. Sections were obtained from two sheep 6 months after the surgical implantation of the nHAP-coated PCL/HAP/β-TCP scaffold and FGF-2-treated BM-MSCs, and from two sheep that received the nHAP-coated PCL/HAP/β-TCP scaffold and BM-MSCs treated with FGF-2 and BMP-2. The presence of bone-specific proteins was verified with FITC green fluorescence. It was found that BM-MSCs treated with FGF-2-treated and treated with FGF-2 and BMP-2, grafted with the nHAP-coated PCL/HAP/β-TCP scaffold into the mandible site, showed the production of both collagen type I and osteocalcin (Figure 10a,b). The average fluorescence intensity graphs showed slight autofluorescence in the negative control for collagen type I (Figure 10c) and even smaller autofluorescence for osteocalcin (Figure 10d).

### 3.11. Cytokine Profile of the Sheep Serum after BM-MSC-Scaffold Grafting

**Figure 11:**
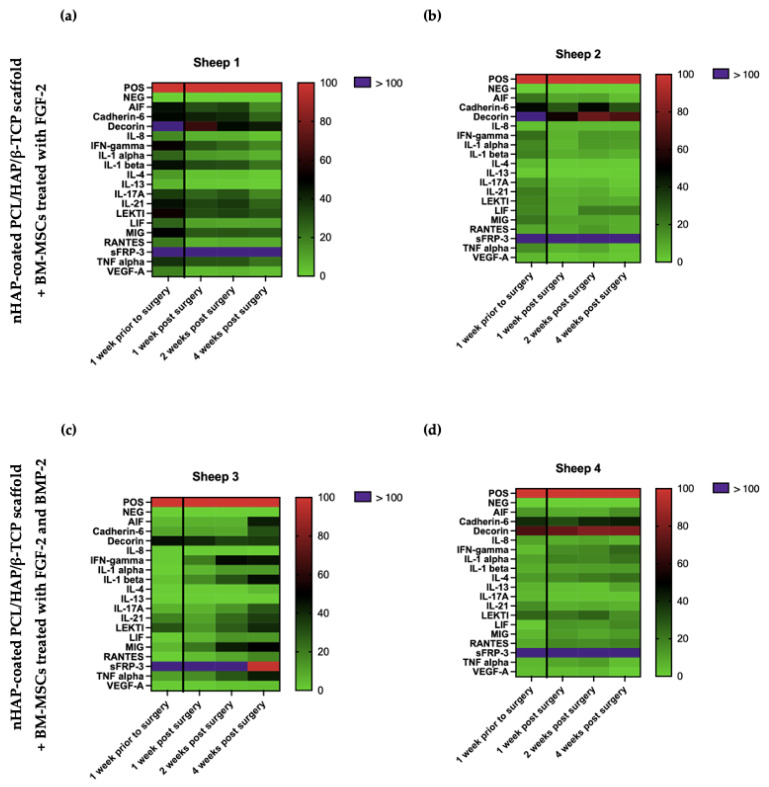
Cytokine profiles of sheep serum 1 week before and 1, 2, and 4 weeks after the transplantation of an nHAP-coated PCL/HAP/β-TCP scaffold and BM-MSCs treated with FGF-2 (**a**,**b**) and FGF-2 combined with BMP-2 (**c**,**d**).

Serum samples were collected from the sheep one week before the BM-MSCs and the nHAP-coated PCL/HAP/β-TCP scaffold grafting and at three time-points post-surgery: one, two, and four weeks, and were assessed for the presence of 18 cytokines using a semi-quantitative cytokine array (Figure 11). Cytokine expression was assessed relative to baseline one week prior to surgery in order to determine the activity of trophic factors promoting osteogenesis and the immune response in sheep after surgery. Two sheep underwent transplantation with the scaffold and FGF-2-treated BM-MSCs (Figure 11a,b) and another two with the scaffold and FGF-2- and BMP-2-treated BM-MSCs (Figure 11c,d). The secreted frizzled-related protein-3 (sFRP-3), which regulates osteoblast differentiation, showed the highest relative expression level, over 100% of the positive control, in all sheep independently from sampling time. However, a slight reduction in sFRP-3 level was observed in sheep 3 (receiving BM-MSC treated with FGF-2 and BMP-2) four weeks after surgery, but still maintaining a high expression of around 100%. Similarly, decorin, which controls fibrillogenesis, also showed a high relative expression in all sheep. It is worth mentioning that the expression of decorin, which is involved in bone formation, decreased in sheep 1 and 2 from over 100% before the implantation to 60–70% at the time-point of four weeks after surgery. A decrease in decorin expression was also observed in the serum from sheep 3 after the implantation. However, in the serum from sheep 4, decorin level increased from about 60% before surgery to about 80% four weeks after the implantation. The apoptosis-inducing factor (AIF), in addition to controlling programmed cell death, also regulates cell proliferation and differentiation. Its level decreased four weeks after surgery, compared to the baseline before surgery, in sheep 1 and 2, which underwent PCL-HA implantation with FGF-2-treated BM-MSCs. Interestingly, the serum from sheep 3 and 4 treated with the nHAP-coated PCL/HAP/β-TCP scaffold and BM-MSCs supplemented with both FGF-2 and BMP-2 showed a higher AIF expression after the implantation than before it. An analysis of the level of inflammatory cytokines, such as interleukin 8 (IL-8), interferon gamma (IFN-gamma), interleukin 1 alpha (IL-1 alpha), interleukin 1 beta (IL-1 beta), interleukin 17A (IL-17A), interleukin 21 (IL-21), monokine induced by interferon gamma/CXCL9 (MIG), and the tumor necrosis factor (TNF-alpha) indicated that the serum from sheep 1 and 2 had slightly elevated levels before surgery, whereas four weeks after it, the level of inflammatory cytokines decreased. Conversely, in the serum from sheep 3, which underwent the transplantation of the scaffold and BM-MSCs treated with FGF-2 and BMP-2, the increase in the relative expression of inflammatory cytokines was observed four weeks after surgery, compared to the baseline before surgery. The serum from sheep 4 showed the lowest expression of inflammatory cytokines at the baseline time-point as well as four weeks post-surgery.

## 4. Discussion

Recently, mesenchymal stem cells have been widely investigated in regenerative medicine for their usefulness in bone defect restoration [35,36,37,38]. Although in vitro studies have provided many promising results, some problems in in vivo studies remain unsolved, such as a limited survival rate of MSCs, maintaining the cells in the injured site, and a low efficacy of differentiation into the specialized cells of the damaged tissue [17]. Consequently, a major challenge for clinical trials involving MSCs is optimizing the cell culture protocol in vitro in order to mimic the natural in vivo MSC environment [39]. For this purpose, we developed a culture of ovine BM-MSCs on a nHAP-coated PCL/HAP/β-TCP scaffold and analyzed their osteogenic differentiation ability when treated with FGF-2 alone or using a combination of two cytokines, FGF-2 and BMP-2. The cells were cultured in 3D conditions to mimic a cell environment resembling native tissue and were supplemented with FGF-2 and BMP-2 to confirm our hypothesis that these proteins could improve the osteogenic potential of MSC in vitro in 3D culture conditions, as demonstrated in our previous study for a 2D culture [25].

Microscopic observations indicated that the ovine BM-MSCs attached to the scaffold structure well and maintained proliferative activity over 21 days of incubation. Interestingly, FGF-2 and BMP-2 affected the spread of cells on the nHAP-coated PCL/HAP/β-TCP scaffold. The presence of FGF-2 in the culture medium efficiently supported the cells to grow between the scaffold bars compared to the control medium without supplements. However, the most beneficial effect on cell aggregation was observed when both FGF-2 and BMP-2 were applied, indicating that BMP-2 in combination with FGF-2 enhanced cell distribution and proliferation on the scaffold. Because the arrangement of the cells between the bars of the scaffold cannot be observed under a light microscope, we additionally observed the MSC culture on the scaffold under a fluorescence microscope after DAPI staining. This approach allowed us to obtain a full overview of cell viability on the surface of the scaffold as well as between its bars. Based on DAPI nucleus staining on day 21 of the BM-MSC culture on the nHAP-coated PCL/HAP/β-TCP scaffold in different culture media, we demonstrated that the ability of the cells to adhere to the scaffold improved when treated with both FGF-2 and BMP-2, compared to the untreated cells. This effect was also confirmed for the standard αMEM and the osteogenic differentiation medium.

To support our microscopic observations of the beneficial effect of FGF-2 and BMP-2 on MSC adhesion and proliferation on the scaffold, we additionally investigated differences in cell attachment to the scaffold according to a culture medium with or without biological factors using the quantitative Picogreen method, as well as cell proliferation and viability using an MTT assay. The number of BM-MSCs on the scaffold was assessed three hours post-seeding. Due to the short incubation time, the adherence capacity of BM-MSCs to the scaffold was analyzed immediately after seeding, which in turn reflected the efficiency of cell deposition on the scaffold before surgery, because the BM-MSCs-scaffold construct was prepared and transplanted into the sheep on the same day. FGF-2-treated ovine BM-MSCs attached to the nHAP-coated PCL/HAP/β-TCP scaffold over twice as effectively as untreated MSCs. Nonetheless, FGF-2 together with BMP-2 increased cell attachment more than three times. Although the microscopic assessment and the Picogreen test showed the best results for FGF-2- and BMP-2-treated cells, the proliferation rate assessed with the MTT was highest for FGF-2 alone-treated BM-MSCs. However, because microscopic observations of cells are inaccurate due to the imperfection of the human eye, it is difficult to draw conclusions about the number of cells based solely on such observations. On the other hand, the Picogreen assay only tests the number of cells 3 h after seeding, providing an insight into cell attachment rather than proliferation. The MTT assay was conducted at four time points over 7 days. However, on day 7 of observation, the proliferation rate for untreated cells and both FGF-2- and BMP-2-treated cells increased considerably, and there were no significant proliferation differences in all groups. Our previous study indicated that ovine BM-MSCs in standard 2D culture conditions proliferated in the αMEM medium; however, the proliferation was slower compared to a conditioned medium with the growth factors FGF2 and BMP-2 (observation was performed for up to 4 days) [25]. Similar effects were observed in this study up to day 5. The rapid increase in cell proliferation on day 7 in all culture conditions, with a high likelihood, indicated the start of osteogenic differentiation processes in the BM-MSCs preconditioned with FGF2 and BMP-2, and consequently, a slow-down in the proliferation of the preconditioned cells but not the untreated cells. These results suggest that the nHAP-coated PCL/HAP/β-TCP scaffold is not toxic for cells in vitro and supports cell proliferation and differentiation. Our previous observations and the present study showed that FGF-2 alone increased cell proliferation of BM-MSCs in both 2D and 3D culture on an nHAP-coated PCL/HAP/β-TCP scaffold better than FGF-2 together with BMP-2 due to the fact that the cells may have already entered the osteogenic differentiation pathway through the synergistic action of FGF-2 and BMP-2 [25]. Our results are consistent with those obtained in a study by Zhang et al. on the osteogenic differentiation enhancement and slower proliferation of BM-MSCs grown on HA scaffolds carrying microspheres with BMP-2 [40]. Moreover, Xu et al. also reported that BMP-2-coated Poly-l-Lactic Acid (PLLA) fibers improved the osteogenic differentiation of MSCs more than their proliferation [41].

The findings that FGF-2 and BMP-2 increase MSC capacity to adhere and proliferate on an nHAP-coated PCL/HAP/β-TCP scaffold have not yet been reported elsewhere. However, Hu et al. demonstrated that FGF-2 and BMP-2 support BM-MSC growth and adhesion on another type of scaffold, i.e., a nanohydroxyapatite and collagen nHAP/COL scaffold [42]. Moreover, the loading of FGF-2 and BMP-2 in composite nanofiber scaffolds has been found to promote the adhesion and proliferation of pre-MC3T3-E1 osteoblasts [43].

In addition to the effect of FGF-2 and BMP-2 on ovine BM-MSCs morphology, adhesion, and proliferation on an nHAP-coated PCL/HAP/β-TCP scaffold, we also investigated their impact on the osteogenic differentiation potential of BM-MSCs in vitro. We focused on matrix mineralization tested with Alizarin Red S staining, ALP activity, and relative expression levels of early osteogenic marker genes: *BMP-2, Runx2, osterix*, and *collagen type I* and late marker genes: *osteocalcin* and *osteopontin*.

Alizarin Red S staining shows the efficiency of the mineralization stage in the osteogenic differentiation of MSCs and has been considered as a marker for calcium compounds common to bone-like structures [44]. Our study showed that the addition of only FGF-2 to the osteogenic differentiation medium did not significantly impact the matrix mineralization of BM-MSCs grown on the nHAP-coated PCL/HAP/β-TCP scaffold. However, cells treated with both FGF-2 and BMP-2 showed a higher intensity of Alizarin Red S staining. The increase in calcium nodule deposition was gradual for all medium conditions, whereas ALP activity significantly increased after 21 days of BM-MSCs incubation on the scaffold. Although ALP is a marker of the early osteogenesis of MSCs, its greatest activity was reported at the last time point of observation after 21 days. This result is consistent with that obtained by Davis et al., who also detected an ALP activity increase over 28 days in BMP-2-treated MSCs incubated on apatite-coated scaffolds [45]. As with Alizarin Red S staining, the strongest impact on ALP activity was reported for FGF-2- and BMP-2-treated cells, suggesting the importance of BMP-2 in osteogenic process control. This finding is consistent with a study by Sun et al., which demonstrated that BM-MSCs grown on PCL/decellularized small intestine submucosa (SIS) scaffold showed a significantly improved osteogenic differentiation capacity when the scaffold contained BMP-2 [46]. Furthermore, Ren et al. demonstrated that a combination of bFGF and BMP-2 synergistically enhanced ALP activity and the calcium mineralization capacity of MC3T3-E1 cells on PLGA/HA nanofiber scaffolds [43].

To confirm the synergistic effect of FGF-2 and BMP-2 on the osteogenic differentiation potential of ovine BM-MSCs grown on an nHAP-coated PCL/HAP/β-TCP scaffold, we also investigated the expression of osteogenesis-related mRNA. As the main osteogenic differentiation characteristics of MSCs are often associated with the upregulation of specific genes in each stage, we focused on early markers such as *Runx2, BMP-2, osterix,* and *collagen type I* and late osteogenic marker genes *osteocalcin* and *osteopontin*. Sheep BM-MSCs grown on an nHAP-coated PCL/HAP/β-TCP scaffold cultured with αMEM supplemented with only FGF-2 or FGF-2 and BMP-2 were compared with those incubated with αMEM medium, which served as a control. At the early time points of 7 and 14 days, no remarkable differences in the relative expression of *Runx2* were detected between the groups. However, after 21 days of incubation, BM-MSCs grown on the nHAP-coated PCL/HAP/β-TCP scaffold treated with FGF-2 and BMP-2 showed a significantly higher expression of *Runx2* than the untreated cells or cells treated with FGF-2 only. Although *Runx2* is an early marker of osteogenesis, in our study, its upregulation was observed at the last time point of observation. This phenomenon may be explained by the fact that osteoblasts are able to enhance the osteogenic differentiation potential of undifferentiated cells in their surroundings, leading to a higher relative expression of *Runx2* in the late phase [47]. A similar effect was reported by Westhauser et al. for MSCs grown on a β-TCP-based scaffold [48]. The expression of another early marker, *osterix,* increased significantly after 7 and 14 days of incubation in FGF-2- and BMP-2-treated ovine BM-MSCs compared to untreated or only FGF-2-treated cells. However, the highest peak was reported on day 21. Our data suggest that *osterix* expression is BMP-2-dependent at an early as well as late stage of osteogenesis, during which still-undifferentiated BM-MSCs are stimulated towards osteogenic differentiation through the autocrine and paracrine mechanisms regulated by already-differentiated cells. Moreover, our study demonstrates that BMP-2 activates *Runx2* expression, which in turn upregulates *osterix* gene expression. The role of BMP-2 in MSC osteogenic differentiation by means of controlling the expression of *Runx2* and *osterix* has already been extensively investigated by other authors [49,50,51]. Interestingly, the expression of *BMP-2* increased over time in all culture conditions. On day 14, its highest relative expression level was reported in MSCs treated with both FGF-2 and BMP-2. However, after 21 days, the highest peak was reported in FGF-2-treated cells. This suggests that BMP-2 autoregulates its own expression. Remarkably, *collagen type I* expression decreased after 14 days of MSC incubation regardless of cytokine stimulation to later slightly increased at day 21; however, its level was still lower than at day 7. Although *collagen I* is considered as a significant factor that supports bone tissue, it was downregulated during the osteogenic differentiation of ovine BM-MSCs grown on the nHAP-coated PCL/HAP/β-TCP scaffold. Nantavisai et al. also reported that *ColI* expression decreased over the course of osteogenic induction in MSC from canine bone marrow and dental pulp [52]. The *Osteocalcin* expression level increased significantly on day 21, as expected; however, its highest peak was reported for FGF-2-treated BM-MSCs, and a lower expression level was observed for both FGF-2- and BMP-2-treated cells. The expression level of the second late osteogenic marker, *osteopontin*, increased slightly over time and was the highest in FGF-2- and BMP-2-treated cells after 21 days of incubation. These results show that FGF-2 plays a crucial role in the osteogenic differentiation of MSCs in 3D culture.

In addition to the impact of FGF-2 and BMP-2 stimulation on the osteogenic potential of ovine BM-MSCs grown on an nHAP-coated PCL/HAP/β-TCP scaffold in vitro, we assessed scaffold biocompatibility and potential for bone regeneration in vivo in a large animal sheep model. At this stage of research, surgical procedure was performed only in four sheep to assess scaffold biocompatibility and to obtain preliminary data on the osteogenic potential of the BM-MSCs-scaffold construct and post-surgical inflammatory response. Immunofluorescence staining of the tissue sections retrieved 6 months post-grafting for osteoblast-specific proteins collagen type I, as an early marker of osteogenic differentiation, and osteocalcin, as a late marker, showed both proteins were present in the site of scaffold-BM-MSCs implantation in all examined sections. Moreover, there were no significant differences in the immunofluorescence staining intensity of collagen type I and osteocalcin between the sections supplemented with BM-MSCs treated with FGF-2-alone or FGF-2 and BMP-2.

Finally, to assess biocompatibility, we monitored the activity of trophic factors involved in osteogenesis and immune response associated with the healing process of the sheep mandible area after the implantation of the scaffold-BM-MSC construct. The cytokine profile in the serum of the sheep that underwent the surgical procedure analyzed by a semi-quantitative cytokine array measuring 18 cytokines did not show any significant differences between the level before grafting (as the baseline) and over four weeks after surgery. Interestingly, sFRP-3 and decorin levels were significantly high in all analyzed samples regardless of the time-point of collection. sFRP-3 promotes the osteogenic differentiation of MSCs by antagonizing non-canonical Wnt signaling [53], whereas decorin is involved in all phases of bone formation, including cell proliferation, matrix mineralization, remodeling, and mineral deposition [54,55]. The results showed that the osteogenic potential was maintained after the implantation of the nHAP-coated PCL/HAP/β-TCP scaffold-BM-MSC, as confirmed by the activity of the osteogenic factors in the sheep serum. In the serum samples from sheep 1 and 2 (which underwent nHAP-coated PCL/HAP/β-TCP scaffold and FGF-2-treated BM-MSC transplantation), inflammatory cytokines, including IL-8, IFN-gamma, IL-1 alpha, IL-1 beta, IL-17A, IL-21, MIG, and TNF-alpha, were found to be at the same or slightly decreased level compared to the pre-operative level. Only the serum from sheep 3 showed an increased level of pro-inflammatory cytokines four weeks after the scaffold-BM-MSC implantation, compared to the baseline. However, there were no clinical signs of inflammation. MSCs are known to not only differentiate into the desired cells but also secrete a variety of bioactive factors with immunomodulatory properties [56]. In this sheep model, MSCs show paracrine activity by affecting immunocompetent cells and modulating the local environment, alleviating the inflammatory response and promoting wound healing.

This preliminary in vivo study showed that BM-MSCs preconditioned with FGF-2 and BMP-2 and implanted on an nHAP-coated PCL/HAP/β-TCP scaffold are biocompatible with sheep tissues and promote bone regeneration. These pilot in vivo observations constitute a foundation for further research and will aid the reconstruction of large bone defects in the mandible region of the sheep model. The in vivo research will be continued with new surgical procedures and a control group of sheep that will undergo scaffold implantation with untreated BM-MSCs to assess the regeneration potential of a scaffold supported with BM-MSCs preconditioned with FGF-2 and BMP-2.

## 5. Conclusions

In summary, our results have demonstrated that an nHAP-coated PCL/HAP/β-TCP scaffold provides a good microenvironment for ovine BM-MSC adhesion and proliferation. Stimulation of the cells with FGF-2 and BMP-2 has a beneficial effect on cell attachment and spread on the scaffold. Our study has shown that simultaneous action of both FGF-2 and BMP-2 increases the osteogenic potential of ovine BM-MSCs grown on the scaffold better than FGF-2 alone. FGF-2 plays a crucial role in MSCs proliferation, whereas BMP-2 influences their osteogenic potential. A tissue engineering construct in the form of an nHAP-coated PCL/HAP/β-TCP scaffold and ovine BM-MSCs preconditioned with FGF-2 and BMP-2 is biocompatible with the sheep tissue environment. We confirmed the biocompatibility of the implanted scaffold-BM-MSCs through the expression of early (collagen type I) and late (osteocalcin) osteogenic proteins and a lack of an elevated level of proinflammatory cytokines. Overall, these data show a promising strategy for clinical application in the repair of large bone defects.

## Figures and Tables

**Table 1:** Primer sequences for qRT-PCR.

Gene	Primer Sequences (5′-3′)	Fragment Size (bp)	Cycles	Tm (°C)	GenBank Accession no.
*GAPDH*	F: GCAAGTTCCACGGCACAG R: GGTTCACGCCCATCACAA	249	40	58	AF030943.1
*BMP-2*	F: ATGGTTTCGTGGTGGAGGTAG R: ACTTGAGGCGTTTCCGCTGTT	210	40	58	AF508028.1
*Runx2*	F: TCGCCTCACAAACAACCA R: AGGGACCTGCGGAGATTA	102	40	53	DY517479.1
*Osterix*	F: CAGCGGCGTGCAGTAAAT R: CTGGGAACGAGTGGGAAAA	240	40	56	BC151270.1
*Collagen type I*	F: CAAGAAGAAGACATCCCACC R: AGATCACGTCATCGCACA	133	40	55	AF129287.1
*Osteocalcin*	F: AGATGCAAAGCCTGGTGATGC R: CTCCTGGAAGCCGATGTGGT	211	40	60	DQ418490.1
*Osteopontin*	F: TCCCACTGACATTCCAACAA R: CTGTGGCATCTGGACTCTCA	196	40	60	AF152416.1

## Data Availability

All data generated or analyzed during this study are included in this article.
